# Trends in gastric cancer mortality 1990–2019 in 36 countries worldwide, with predictions to 2025, and incidence, overall and by subtype

**DOI:** 10.1002/cam4.5685

**Published:** 2023-02-23

**Authors:** Giulia Collatuzzo, Claudia Santucci, Matteo Malvezzi, Carlo La Vecchia, Paolo Boffetta, Eva Negri

**Affiliations:** ^1^ Department of Medical and Surgical Sciences University of Bologna Bologna Italy; ^2^ Department of Clinical Sciences and Community Health University of Milan Milan Italy; ^3^ Stony Brook Cancer Center Stony Brook University Stony Brook New York USA

**Keywords:** cardia, gastric cancer, incidence, mortality, trends

## Abstract

**Background:**

Gastric cancer (GC) incidence is declining heterogeneously worldwide. We aimed to calculate updated mortality trends for GC.

**Methods:**

We investigated time trends for selected countries using the World Health Organization database. We computed age‐standardized mortality rates (ASMR) per 100,000 persons over the 1990–2019 period. We reported rates for the 2010–2014 and 2015–19 calendar periods, and the corresponding percent changes. We used joinpoint regression analysis to identify changes in the slope of mortality trends, and predict the number of deaths and rates for 2025. We also reported 2008–2012 incidence rates of cardia and noncardia GC.

**Results:**

Mortality trends from GC have been favorable since 1990 for all countries analyzed and the European Union (EU 27), in both sexes and all ages. GC mortality is predicted to decline in all countries for both sexes, except for French and US women aged 35–64 years, and Canadian men aged 35–64. The highest proportions of cardia GC were observed in Northern and Central Europe while the lowest ones in Southern and Eastern Europe. Elsewhere, the highest proportions were registered in countries with low incidence and mortality rates, whereas high‐mortality countries showed lower proportions of cardia GC.

**Conclusion:**

Observed and predicted GC mortality trends declined in most countries in both sexes, with few exceptions, likely due to the control of GC risk factors, in particular *Hp* infection.

## Graphical abstract

Age‐standardized mortality rates from gastric cancer per 100,000, 2015‐2019, in men and women in selected countries

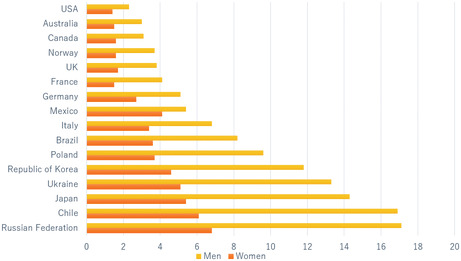

## 1 INTRODUCTION

Gastric cancer (GC) was the leading cancer worldwide until 1975,^1^ it still accounts for over one million new cases (https://gco.iarc.fr), being the fourth cause of cancer mortality in 2020.^2^ GC prognosis is poor, the disease is often diagnosed at an advanced stage where aggressive surgery is needed.^3^ Consequently, prevention to control the burden of GC is crucial. Infection with *Helicobacter pylori* (*Hp*) is the major cause of GC and is widespread globally, with differences by country, socioeconomic status (SES), and birth cohort.^4^ Infection prevalence has been declining progressively over the years, largely thanks to water sanitation.^5^ Tobacco smoking, alcohol consumption, unbalanced diet, and other factors associated with low SES are major concomitant factors, while occupational and genetic factors play an additional role.^6^ The burden of GC is larger among individuals with low SES and in low‐ and middle‐income countries.^7^ Asian countries experience a heavier burden of GC compared to other regions, with Mongolia, Japan and the Republic of Korea being the countries with the highest GC incidence in 2020.^8^ Cardia and noncardia GC subtypes (this latter including fundus, corpus, large and lesser curvatures and antrum) representing 15% and 75% of cases, respectively, can be identified based on the proximal or distal origin of the neoplastic lesions.^9^ Given that the anatomical locations are associated with different risk factors, with cardia GC sharing risk factors with esophageal adenocarcinoma such as obesity and gastroesophageal reflux while noncardia GC is strongly associated with *Hp* infection, GC shows different patterns across subgroups of the population and geographical areas. Conversely, the histological distinction of GC into intestinal and diffuse type does not translate into heterogeneity in GC epidemiology.^3^ Upper‐endoscopy is the gold standard for GC diagnosis, which is based on biopsy analysis.^10^

We aimed at estimating and interpreting the mortality trends of GC worldwide between 1990 and 2019, with emphasis on the last 10 years, focusing on sex and age differences. We also showed the proportion of cardia to noncardia incidence subtypes. Moreover, we reported the projected mortality rates and deaths for the 2025 calendar year.

## 2 MATERIALS AND METHODS

### 2.1 Mortality

Using the WHO mortality database,^11^ we analyzed official numbers of certified deaths from GC coded as C16 according to the 10th Revision of the International Classification of Disease,^12^ in selected countries worldwide. The European Union as a whole was defined as the 27 member states, EU (27), as of January 2020. We restricted our analyses to countries with high‐mortality coverage (i.e., over 90%), high data quality, and more than 5 million inhabitants identifying 36 countries worldwide plus the EU.^13^ We extracted estimates of resident populations from the same WHO database^11^ for European and Australasian countries and from the Pan American Health Organization (PAHO) database for American countries.^14^ When population data were missing, we retrieved them from EUROSTAT^15^ or the United Nations (UN) Population Division^16^ databases. Thus, we derived figures for the calendar periods from 1990 up to the most recent available year.

For each country, calendar year, and sex, we calculated 5 years' age‐specific rates and the age‐standardized mortality rates (ASMRs) per 100,000 person‐years, using the world standard population,^17^ at all ages and for the 35–64 year age group. We then fitted joinpoint regression models^18^ from 1990 until the most recent available year, for the most populous (≥10 million) countries and the EU (27), to identify significant changes in the linear slope (on a log scale) of ASMRs. For each identified linear segment, we estimated annual percent change (APC) and, for the entire study period, the average annual percent change (AAPC).^19^, ^20^

For countries with ≥20 million inhabitants (i.e., 15 countries plus the EU as a whole), we also predicted the number of deaths for the year 2025, by fitting a Poisson joinpoint regression model, with a maximum of 5 possible joinpoints^19^ to the number of deaths in each 5‐year age group and running a linear regression over the most recent trend segment identified, the joinpoint model was restricted to identifying models with the final segment having at least 5 data points. Using the matrices of predicted age‐specific death counts and predicted populations, we computed predicted ASMRs and their 95% prediction intervals (PIs). The predicted populations were extracted using the PAHO database for North American and Latin American countries, the EUROSTAT database for European countries, and the UN database for Asian and Oceanian countries.

### 2.2 Incidence

We obtained incidence data for GC for the 2008–2012 calendar period from the Cancer Incidence in Five Continents database, volume XI.^21^ For countries with more than one cancer registry, we aggregated data to ensure the largest geographic coverage, and we restricted analyses to the longest common calendar period between registries. We presented, separately by sex, the number of cardia (C16.0) and noncardia (C16.1–6) incident cases of GC in various countries worldwide, in order to show the proportion of cardia cases.

We only considered publicly available data, so no clearance by Ethical Committee was necessary. For statistical analyses, we used R version 4.1.3 (R Development Core Team, 2017), SAS version 9.4 (SAS Institute Inc.), and Joinpoint Regression Program version 4.9.1.

## 3 RESULTS

**FIGURE 1:**
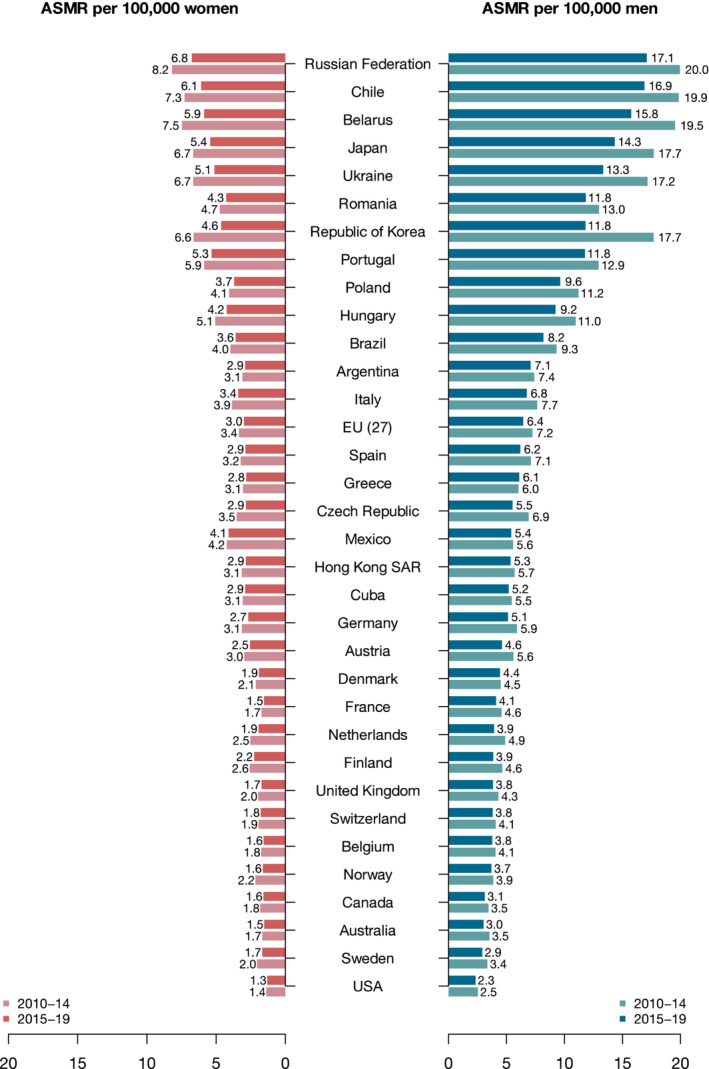
Bar plots of age‐standardized mortality rates (ASMR) from gastric cancer, per 100,000 men and women, in selected countries worldwide, in 2010–14 and 2015–19.

Table 1 shows the annual average number of deaths and ASMRs from GC per 100,000 person‐years of all ages and both sexes in selected countries worldwide and the EU (27), for the periods 2010–14 and 2015–19, together with the corresponding percent changes. The ASMRs ordered from the highest to the lowest one during the most recent period displayed by bar plots for men and women are also shown in Figure 1. Except for Greek males, the change in rates was favorable in all countries and both sexes. During 2015–2019, the highest male rates were reported in the Russian Federation, Chile, and Belarus with rates higher than 15/100,000, followed by Japan, Ukraine, Romania, South Korea, and Portugal with rates higher than 10/100,000. The lowest rates were observed in the United States, Sweden, Australia, and Canada with rates around 2–3/100,000 males. Similarly for females, the Russian Federation, Chile, Belarus, Japan, Portugal, and Ukraine showed the highest ASMRs during the 2015–2019 calendar period (rates varied from 5.1 to 6.8/100,000). The lowest ASMRs were registered in the United States, Australia, Canada, Belgium, Norway, Sweden, and Finland; with rates around 1.5–1.7/100,000 females. Corresponding figures for truncated rates in the 35–64 years age group are reported in Table S1 and Figure S1. The change in rates from 2010–2014 to 2015–2019 was favorable in all countries considered and both sexes, except for Denmark (+3.3%), Greece (+5.9%), Norway (+10.0%), Switzerland (+1.4%), and Mexico (+0.4%) in males and the United States (+1.0%) in females, where the rates were higher in the most recent period. Table S2 and Figure S2 report ASMRs among order adults aged 65 or more. Among the elderly, the change in rates from 2010–2014 to 2015–2019 was favorable in all countries considered and both sexes. The ranking was similar to the all ages one with some differences, that is, Chile and Japan were the first‐ranking countries for the elderly and the second and fourth one at all ages. This indicates steeper declines in young and middle age in those countries as compared to Russia and other eastern European countries.

**TABLE 1:** Annual average deaths and age‐standardized (world population) mortality rates from gastric cancers per 100,000 person‐years in 2010–14 and 2015–19 for both sexes, along with the corresponding change in rates.

	Men					Women				
	Annual average deaths 2010–14	ASMR 2010–14	Annual average deaths 2015–19	ASMR 2015–19	% change 2015–19 vs. 201,014	Annual average deaths 2010–14	ASMR 2010–14	Annual average deaths 2015–19	ASMR 2015–19	% change 2015–19 vs. 2010–14
Europe										
Austria	499	5.60	447	4.61	−17.7	384	2.96	334	−13.9	
Belarus	1219	19.53	1064	15.75	−19.4	823	7.47	679	5.86	−21.6
Belgium	468	4.06	457	3.77	−7.1	297	1.75	269	1.57	−10.3
Czech Republic	664	6.91	594	5.52	−20.1	470	3.52	408	2.85	−19.0
Denmark	254	4.51	274	4.45	−1.3	144	2.14	140	1.91	−10.7
Finland	265	4.64	244	3.86	−16.8	197	2.57	179	2.25	−12.5
France	2959	4.58	2848	4.10	−10.5	1657	1.72	1531	1.54	−10.5
Germany	5675	5.91	5270	5.13	−13.2	4221	3.14	3698	2.68	−14.6
Greece	799	6.04	819	6.10	1.0	510	3.06	495	2.83	−7.5
Hungary	947	10.97	845	9.23	−15.9	709	5.07	618	4.24	−16.4
Israel	287	5.48	288	4.76	−13.1	200	3.08	196	2.69	−12.7
Italy	5728	7.65	5489	6.75	−11.8	4115	3.85	3882	3.40	−11.7
Kyrgyzstan	417	23.58	461	22.23	−5.7	194	8.01	216	7.78	−2.9
Netherlands	836	4.89	769	3.94	−19.4	539	2.53	452	1.93	−23.7
Norway	191	3.87	197	3.70	−4.4	140	2.16	113	1.63	−24.5
Poland	3436	11.21	3271	9.63	−14.1	1845	4.06	1812	3.70	−8.9
Portugal	1406	12.94	1370	11.76	−9.1	932	5.87	900	5.33	−9.2
Romania	2282	12.98	2177	11.84	−8.8	1182	4.74	1115	4.27	−9.9
Russian Federation	18,481	19.95	16,914	17.09	−14.3	13,886	8.19	12,168	6.76	−17.5
Serbia	653	9.11	603	8.07	−11.4	375	4.15	324	3.61	−13.0
Spain	3452	7.11	3285	6.19	−12.9	2208	3.23	2074	2.88	−10.8
Sweden	371	3.36	339	2.92	−13.1	267	2.05	222	1.67	−18.5
Switzerland	331	4.07	340	3.81	−6.4	202	1.94	205	1.78	−8.2
Ukraine	5360	17.16	4160	13.34	−22.3	3427	6.65	2620	5.12	−23.0
United Kingdom	3003	4.30	2842	3.84	−10.7	1770	1.96	1592	1.72	−12.2
EU (27)	33,010	7.24	31,533	6.44	−11.0	21,672	3.36	20,234	2.99	−11.0
America										
Argentina	1826	7.39	1901	7.08	−4.2	1056	3.10	1069	2.91	−6.1
Brazil	8825	9.32	9350	8.19	−12.1	4905	3.97	5257	3.59	−9.6
Chile	2178	19.85	2186	16.90	−14.9	1104	7.28	1085	6.08	−16.5
Cuba	518	5.46	547	5.19	−4.9	338	3.08	350	2.90	−5.8
Mexico	2912	5.57	3267	5.42	−2.7	2588	4.23	2834	4.10	−3.1
Canada	1153	3.45	1228	3.14	−9.0	753	1.81	738	1.59	−12.2
USA	6688	2.54	6760	2.34	−7.9	4551	1.38	4548	1.31	−5.1
Australasia										
Australia	729	3.53	712	3.03	−14.2	420	1.67	422	1.53	−8.4
Hong Kong SAR	397	5.70	419	5.35	−6.1	266	3.15	268	2.86	−9.2
Japan	32,279	17.70	29,813	14.33	−19.0	16,847	6.65	15,594	5.43	−18.3
Republic of Korea	6137	17.69	5206	11.81	−33.2	3301	6.63	2833	4.63	−30.2

Abbreviation: ASMR, age‐standardized mortality rates using the world standard population.

**FIGURE 2:**
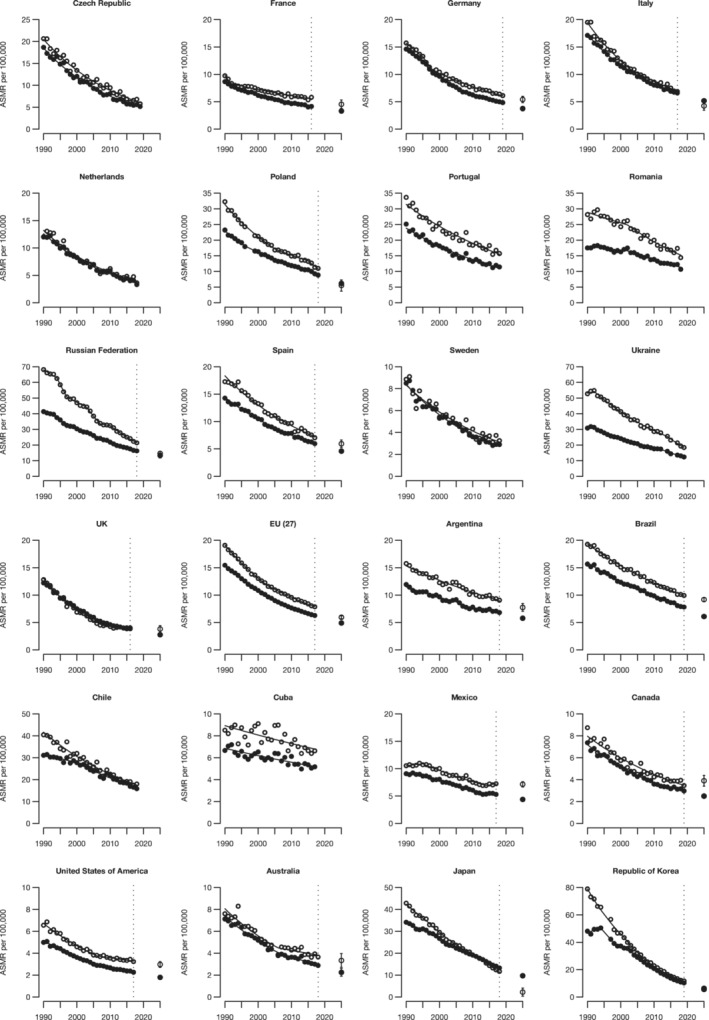
Annual age‐standardized (World standard population) mortality rates per 100,000 males from gastric cancer in major countries (>10 million inhabitants)worldwide and the EU (27), the resulting joinpoint regression models and predicted rates (only for countries with >20 million inhabitants and the EU)for the year 2025 with 95% prediction intervals. The Y axes in the figure (Deaths per 100,000) are not uniform. 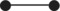 All ages 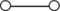 35‐64 years

**FIGURE 3:**
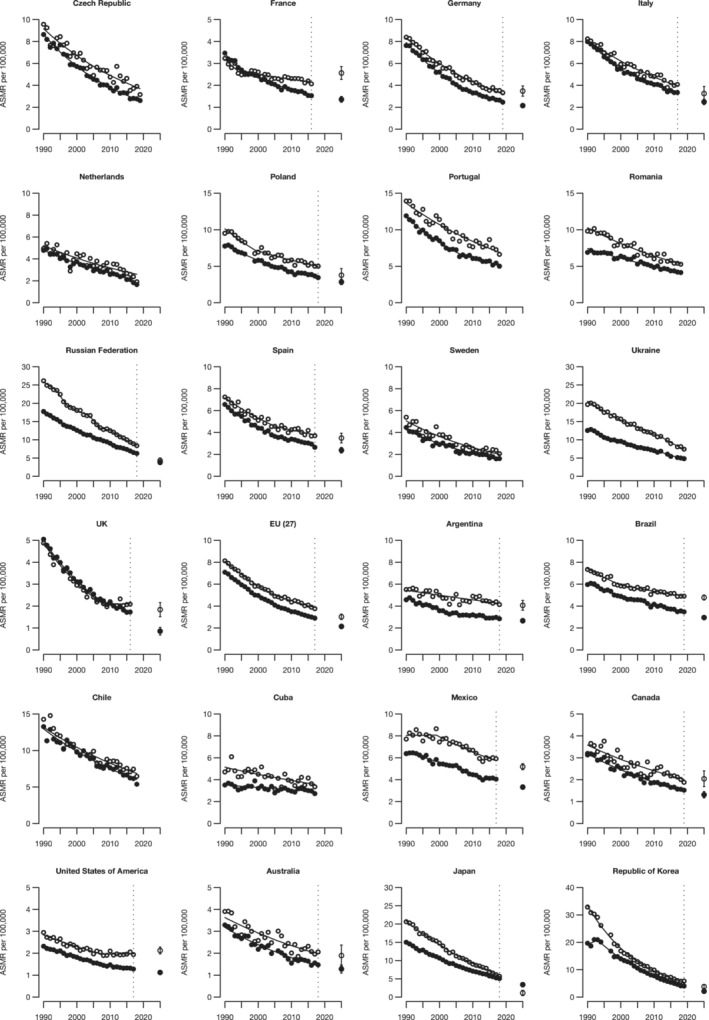
Annual age‐standardized (World standard population) mortality rates per 100,000 females from gastric cancer in major countries (>10 million inhabitants) worldwide and the EU (27), the resulting joinpoint regression models and predicted rates (only for countries with >20 million inhabitants and the EU) for the year 2025 with 95% prediction intervals. The Y axes in the figure (Deaths per 100,000) are not uniform. 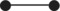
All ages 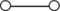
35‐64 years

Figure 2 shows the trends in ASMRs from GC at all ages (full dots) and 35–64 years (empty dots) along with the corresponding joinpoint models (lines) for 23 selected countries worldwide and the EU‐27, over the study period among males. The corresponding figures for females are shown in Figure 3. Results from the joinpoint analyses are reported in Tables S2 and S3, respectively, for males and females. For the largest countries and the EU (27), the predicted ASMRs at all ages and 35–64 years for the calendar year 2025 with the corresponding 95% PIs are also shown. Detailed results for the 2025 predicted deaths and ASMRs are reported in Table 2. Male ASMRs resulted in a decrease since 1990 in all countries considered and the EU (27) and for both all ages and truncated 35–64 years. The Republic of Korea showed the highest decrease over the considered period, with an AAPC of −5.2 for all ages. Czech Republic, Netherlands, Sweden, and the United Kingdom reported moderate decreases (AAPCs greater than −4% per year) too, while Greece, Argentina, Cuba, and Mexico reported less consistent but still significant decrements (<2% per year). The EU (27) reported a significant decrease over time, with an AAPC ‐3.3. Results for males aged 35–64 years were similar to those for all ages.

**TABLE 2:** Number of predicted deaths and mortality rates from gastric cancer, for both sexes, for the year 2025 and comparison figures for 2015–2019, for selected (>20 million inhabitants) countries worldwide and the EU as a whole, with 95% prediction intervals.

	Men					Women				
	Observed mean number of deaths 2015–19	Predicted number of deaths 2025 (95% PI)	Observed ASMR 2015–19	Predicted ASMR 2025 (95% PI)	% difference 2025 versus 2015–19	Observed mean number of deaths 2015–19	Predicted number of deaths 2025 (95% PI)	Observed ASMR 2015–19	Predicted ASMR 2025 (95% PI)	% difference 2025 versus 2015–19
Europe										
France										
All ages	2848	2620 (2450–2800)	4.1	3.32 (3.03–3.6)	−19.1	1531	1180 (1000–1370)	1.54	1.36 (1.23–1.49)	−11.8
35–64 years	787	630 (520–750)	5.58	4.55 (3.77–5.33)	−18.5	310	370 (330–410)	2.13	2.56 (2.27–2.86)	20.3
Germany										
All ages	5270	4440 (4040–4840)	5.13	3.75 (3.45–4.06)	−26.8	3698	2980 (2670–3300)	2.68	2.15 (1.96–2.34)	−19.8
35–64 years	1328	1210 (1110–1310)	6.49	5.42 (4.85–5.99)	−16.5	704	690 (600–770)	3.54	3.48 (3.01–3.95)	−1.8
Italy										
All ages	5489	5090 (4830–5340)	6.75	5.17 (4.81–5.54)	−23.3	3882	3310 (3100–3520)	3.4	2.49 (2.24–2.73)	−26.8
35–64 years	997	640 (520–750)	7.11	4.25 (3.48–5.02)	−40.2	602	520 (420–620)	4.1	3.25 (2.6–3.9)	−20.7
Poland										
All ages	3271	2540 (2310–2770)	9.63	6.16 (5.49–6.83)	−36.1	1812	1590 (1440–1750)	3.7	2.84 (2.48–3.21)	−23.2
35–64 years	1063	450 (300–590)	12.04	5.51 (3.72–7.3)	−54.2	470	320 (240–400)	5.12	3.77 (2.87–4.67)	−26.3
Russian Federation										
All ages	16,914	15,040 (13000–17,070)	17.09	13.29 (11.7–14.88)	−22.2	12,168	7610 (5970–9250)	6.76	3.84 (3.19–4.48)	−43.2
35–64 years	7178	4440 (4040–4850)	23.17	14.63 (13.25–16.01)	−36.9	3465	1600 (1340–1850)	9.16	4.32 (3.61–5.02)	−52.9
Spain										
All ages	3285	2920 (2710–3140)	6.19	4.58 (4.23–4.94)	−25.9	2074	1750 (1580–1930)	2.88	2.39 (2.13–2.64)	−17.2
35–64 years	780	710 (630–790)	7.45	5.94 (5.24–6.63)	−20.3	414	410 (360–460)	3.85	3.5 (3.04–3.95)	−9.2
UK										
All ages	2842	2310 (2120–2510)	3.84	2.76 (2.49–3.04)	−28	1592	790 (640–930)	1.72	0.86 (0.68–1.03)	−50.2
35–64 years	543	560 (460–650)	4.02	3.81 (3.2–4.42)	−5.2	284	270 (230–320)	2.08	1.84 (1.52–2.16)	−11.6
EU (27)										
All ages	31,533	28,440 (27740–29,140)	6.44	4.9 (4.76–5.05)	−23.9	20,234	15,840 (15060–16,610)	2.99	2.14 (2.04–2.25)	−28.3
35–64 years	8239	6390 (6100–6670)	8.1	5.95 (5.68–6.21)	−26.6	4061	3220 (2950–3480)	3.91	3.02 (2.77–3.27)	−22.7
America										
Argentina										
All ages	1901	1960 (1860–2060)	7.08	5.78 (5.48–6.08)	−18.4	1069	1170 (1080–1260)	2.91	2.66 (2.45–2.87)	−8.5
35–64 years	657	610 (550–670)	9.43	7.75 (7.03–8.46)	−17.8	324	340 (310–380)	4.29	4.07 (3.62–4.52)	−5.2
Brazil										
All ages	9350	9990 (9720–10,250)	8.19	6.09 (5.92–6.26)	−25.6	5257	5860 (5620–6100)	3.59	2.95 (2.83–3.08)	−17.7
35–64 years	3665	3890 (3760–4030)	10.35	9.18 (8.86–9.51)	−11.3	1971	2220 (2110–2330)	4.99	4.79 (4.56–5.02)	−4.1
Mexico										
All ages	3267	3790 (3620–3960)	5.42	4.38 (4.19–4.58)	−19.1	2834	3280 (3160–3410)	4.1	3.32 (3.19–3.45)	−19.0
35–64 years	1299	1660 (1540–1770)	7.16	7.15 (6.66–7.64)	−0.1	1168	1310 (1240–1370)	5.93	5.18 (4.91–5.45)	−12.7
Canada										
All ages	1228	1290 (1200–1370)	3.14	2.49 (2.3–2.69)	−20.6	738	720 (640–800)	1.59	1.31 (1.17–1.46)	−17.4
35–64 years	329	350 (300–390)	3.8	3.9 (3.41–4.39)	2.7	179	180 (150–210)	2.06	2.04 (1.68–2.4)	−0.9
USA										
All ages	6760	6640 (6340–6940)	2.34	1.79 (1.7–1.89)	−23.3	4548	4420 (4190–4650)	1.31	1.12 (1.06–1.18)	−14.3
35–64 years	2332	2160 (1980–2340)	3.33	2.97 (2.7–3.24)	−10.9	1384	1490 (1390–1590)	1.98	2.12 (1.97–2.28)	7.3
Australasia										
Australia										
All ages	712	670 (580–750)	3.03	2.25 (1.9–2.6)	−25.8	422	410 (370–460)	1.53	1.28 (1.09–1.47)	−16.3
35–64 years	185	180 (150–210)	3.78	3.34 (2.71–3.96)	−11.7	106	100 (80–130)	2.1	1.89 (1.42–2.37)	−9.8
Japan										
All ages	29,813	25,230 (24280–26,170)	14.33	9.71 (8.99–10.43)	−32.3	15,594	13,520 (12930–14,100)	5.43	3.4 (3.02–3.79)	−37.3
35–64 years	3656	490 (0–1050)	12.9	2.24 (0.38–4.1)	−82.6	1735	230 (30–440)	6.34	1.13 (0.39–1.86)	−82.2
Republic of Korea										
All ages	5206	3660 (3390–3930)	11.81	5.52 (5.02–6.02)	−53.3	2833	1950 (1750–2160)	4.63	2.13 (1.78–2.49)	−54.0
35–64 years	1746	1000 (840–1150)	13.31	6.34 (5.16–7.52)	−52.4	781	550 (460–630)	6.29	3.83 (3.14–4.53)	−39.1

Abbreviations: ASMR, age‐standardized mortality rates using the world standard population; PI, prediction interval.

The Republic of Korea reported the steepest fall in trend over time in females too (AAPC ‐5.2 for all ages). Belgium, the Czech Republic, and the United Kingdom showed favorable trends over time as well (AAPC around −4). Greece, Argentina, Brazil, Cuba, and Mexico had the smallest AAPCs—lower than 2—but still significant. The EU (27) showed a decrease over time with an AAPC of −3.3. Corresponding figures for females aged 35–64 were consistent with the decrease observed at all ages, albeit smaller.

Predicted rates for the year 2025 are downwards for all countries and the EU (27), for both sexes, and for all ages and adults aged 35–64, except for females aged 35–64 in France (% difference in rates versus 2015–19: +20.3) and in the United States (+7.3%), and for males aged 35–64 in Canada (+2.7%); however, these populations had comparatively low rates. Likewise, the predicted number of deaths in 2025 was favorable for all countries, sexes and ages except for Argentina, Brazil, Mexico, and the United States, for which the predicted deaths are higher than the ones observed in 2015–2019, due to population aging and growth. The most favorable predicted rates for 2025 are those in the Republic of Korea (% difference in rates versus 2015–19: −53.3 and −54.0, respectively, for men and women), followed by those calculated in Japan (−32.3% and −37.3%, respectively, for men and women). In the EU (27), the ASMR is predicted to decline in men and women, with, respectively, a −23.9% and −28.3% decrease from 2015–19 to 2025 for all ages and −26.6% and −22.7% for adults aged 35–64 years.

**FIGURE 4:**
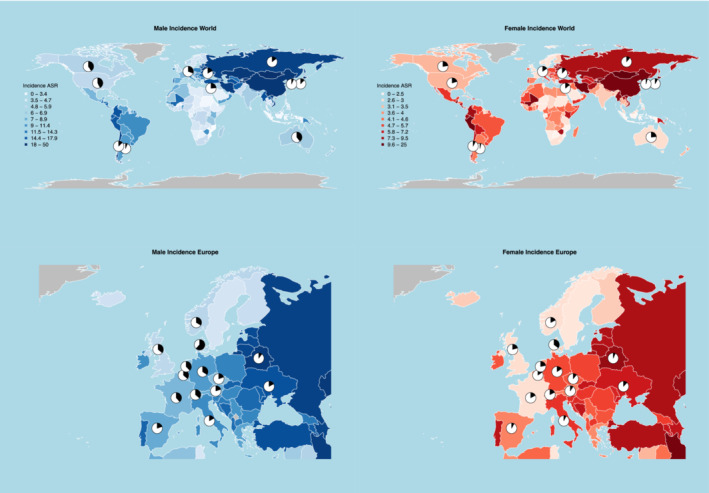
Gastric cancer age‐standardized (World standard population) incidence rate estimates for the year 2020 from Globocan in males and females, proportion of cardia (black) and noncardia (white) incident cancers from Cancer Incidence in 5 Continents Volume XI.

Figure 4 gives the proportion of cardia and noncardia GC in men and women, respectively, for selected countries from Europe and other countries worldwide. For men, among European countries, the proportion of cardia ranged between 10.3% in Belarus and 62.0% in Denmark, being higher in Northern and Central Europe and lower in Southern and Eastern Europe (Table S5). Among other countries worldwide, the highest male proportion of cardia cancers (around 40%) was observed in countries with low incidence and mortality rates such as Canada, the United States, and Australia, while those with high rates, such as Argentina and the Republic of Korea had the lowest proportion (around 6%) of cardia subtype. Among women, a similar geographic pattern was observed (Table S6). In Europe, the lowest female proportion of cardia cases was observed in Belarus (6.9%), followed by Italy (7.4%), Spain (8.2%), and Austria (9.4%), while the highest proportion was observed in Denmark (35.1%). Worldwide, Argentina and the Republic of Korea showed the lowest proportions of cardia cases (respectively, 3.5 and 4.8%) while Canada, the United States, and Australia registered the highest ones (over 20%) among women.

## 4 DISCUSSION

Our results show a declining GC mortality trend, observed since the 1990 s has continued in the 2010 s in all countries. The decline in mortality was more pronounced in countries with higher rates, such as Japan and Korea, compared to countries with lower rates, such as the United States and Canada, consistently with previous results.^22^, ^23^ A strong reduction in GC death rates was also observed in the Russian Federation, Poland, and Italy. During the most recent calendar years, we observed the lowest rates of GC mortality around or below 2/100,000 (for men in United States, and for women, in several northern Europe countries, Australia and North America). A further marked decline is predicted in Japan and South Korea for both sexes up to 2025, while the United States and Canada are expected to experience less favorable trends, including a possible increase in young age groups. Our predictions for 2025 estimate mortality rates in women in low‐risk countries, such as the United Kingdom and the United States, in the order of 1/100,000/year or lower, the conventional threshold for the definition of a rare cancer. This raises interesting questions on whether further reduction could be achieved and what the base incidence of noncardia GC in the absence of *Hp* infection could be. The first symptoms of GC such as dyspepsia and early satiety largely overlap with benign conditions such as gastritis and reflux, while more severe symptoms like pain, weight loss, and hematemesis/anemia appear late.^9^ Indeed, GC is mainly diagnosed at stages III and IV, when survival is poor.^3^ According to Arnold and coauthors, about half of the GC cases diagnosed in Canada, Ireland, Denmark, and the United Kingdom in 2012–2014 belong to stage IV disease.^23^ The authors also described a geographical variation in GC survival for patients with diagnosis of localized disease (e.g., 94.3% in New Zealand and 75.5% in Australia at 1 year and 86.5% in New Zealand and 59.9% in the United Kingdom at 3 years from diagnosis). Smaller differences emerged when comparing survival from distant disease, ranging from 26.6% to 20.7% at 1‐year and from 8.0% to 3.8% at 3‐year postdiagnosis.^23^

Endoscopy and multiple biopsy sampling are the gold standard for GC diagnosis. Also, endoscopic ultrasound helps in identifying wall infiltrations, while endoscopic mucosal resection and endoscopic mucosal dissection provide reliable staging information, allowing in addition the treatment of superficial early‐stage lesions. The endoscopy is subject to the compliance of the patients and the availability of both a trained specialist and an endoscope and endoscopy room. Indeed, it is an invasive and expensive test, and is limitedly available in less affluent countries.^10^ Despite surgical resection may be curative, most patients relapse. The most recent ESMO Clinical Practice Guidelines indicate that stage IB‐III disease requires radical gastrectomy.^24^ Nodal dissection accompanying radical gastrectomy is indicated to cover at least 15 lymph nodes according to the AJCC/UICC TNM, which again implies the availability of large upper‐GI surgical units and highly specialized peri‐operative care centers, which are more common in Asia than in other countries.^24^ Preoperative and postoperative chemotherapy is recommended in all ≥IB resectable GC, possibly including fluoropyrimidine, a platinum agent and docetaxel.^24^ When focusing on locally advanced unresectable or metastatic disease, the prognosis is very poor, with less than 1 year survival.^24^ However, combined chemotherapy has been demonstrated to be more effective than single‐agent chemotherapy or supportive care only. Moreover, survival improvement has been reached by using chemotherapy combined with immunotherapy (nivolumab or trastuzumab).^24^

Part of the mortality decline is due to screening programs and early diagnosis in high‐incidence countries like Japan and Korea. Nearly three‐quarters of new GC cases occur in Asia, but large differences can be found when observing GC trend in Asian countries. A recent study highlighted how the different socioeconomic development, the historical background of cancer control programs explain the different trends in incidence by birth cohorts, where Japan underwent a decline in younger cohorts while China did not.^22^ These authors attributed this difference primarily to the high‐quality cancer control policies undertaken in Japan 22 years earlier than in China. Our results corroborate previous analyses focused on GC trends during 1980–2011, and identified a decrease in GC mortality which was more pronounced for noncardia cancer in non‐Asian countries.^22^ The authors interpreted these results as the progress in *Hp* infection control matched with a birth‐cohort effect characterized by lower prevalence of infection in subsequent generations, causing a lower rates of noncardia GC cases. Also, they described a gradually smaller decline in GC mortality trends in US and predicted no further reduction for France after 2015.^22^

Our findings also agree with the projections to 2040 made in a recent study, which described the estimated incidence and mortality of GC worldwide to be particularly high in Asia and in men, with about two thirds of all cases occurring in the male sex.^23^

When considering subtypes, noncardia is responsible for more cases and, assuming comparable case fatality between the two types,^25^ it likely causes more deaths than cardia GC. Noncardia GC is more frequent than cardia in most countries, with a ratio between 2:1 and 3:1.^26^ Diet and obesity as well as gastroesophageal reflux disease are implicated in the increasing number of cardia cases in US and EU countries.^27^, ^28^ In addition, the ratio reflects past (given 20–30 years of latency for cancer development) prevalence of *Hp*. The high prevalence of infection is responsible for the high ratio in South America, East Asia, and Southern and Eastern Europe.

When looking at the numbers of cardia GC, possible anatomical location misclassification should be taken into account. Indeed, the increased number of cancers of the esophageal junction and of the cardia in cancer registries may be partially due to differences in tumor classification because of discrepancies in the anatomic subsite classification. This discrepancy was observed in a study based on Swedish Cancer Registry, where the authors also noticed a bidirectional misclassification between cancer of the lower esophagus and cancer of the gastric cardia.^29^ Another study also based on Swedish Cancer Registry estimated that true cardia GC incidence could be up to 45% higher or 15% lower than that reported, possibly explaining the increasing number of cardia GC cases in the country.^30^ A subsequent analysis on Cancer Registry of the Swiss Canton of Vaud over the period from 1976 and 1997 aimed at interpreting the reported increasing incidence in cardia GC, did not find an absolute increase in cardia GC cases, but rather a decrease of noncardia GC cases, which resulted in an increase in the proportion of cardia GC.^31^ However, as noted by several authors,^32^, ^33^ the classification of upper GI cancers remains problematic given the lack of a universally accepted and clearly reproducible anatomic landmark separating the gastric cardia from the lower esophagus, and even when the landmarks are defined, cancer often destroys the anatomy of the organ where it occurs, making landmarks become unrecognizable.

Based on our data, the reduction in GC mortality is similar in both sexes, with few exceptions (e.g., Denmark, Greece, and Norway, where declining rates were greater among women), although GC incidence and mortality remain higher in men. Previous studies explained this difference with the distribution of smoking habits and *Hp* infection, which are higher in men than in women: Countries where *Hp* infection shows larger gender gaps (e.g., USA and EU) have a stronger gender difference in mortality rates for GC as well.^34^, ^35^

It will be interesting to see if in countries where cardia GC and its risk factors are not so frequent (e.g., Asian countries) there will be a further decrease in GC overall, because of the low incidence of cardia coupled with a continuous decrease in noncardia GC. Whether the incidence of cardia GC will increase, following the increasing prevalence of its risk factors such as obesity^48^ remains an open question.

For some countries, including the United Kingdom, Brazil, Mexico, Canada, and the United States, the overall reduction in GC mortality was less pronounced among younger subjects, furthermore rising incidence rates in young adults have been reported recently.^26^, ^34^, ^35^, ^36^, ^37^ Age‐cohort effects would suggest a lower incidence of GC among the new generations, due to the birth‐cohort effect of *Hp* infection^38^—well known to be less prevalent in the young^39^—as well as smoking habits, declining progressively in subsequent birth cohorts.^40^ These results are consistent with the findings of Wang and collaborators, who calculated GC incidence rates in the United States between 1999 and 2013: New GC cases have been reported to overall diminish, except for patients diagnosed under 50 years of age.^41^ Moreover, the incidence of advanced noncardia cases has been observed among under‐50 years old Hispanics.^42^ These elements could justify the smaller decrease in GC mortality in the young population. The lower reduction in the young could also be referred as the overall small incidence rate in this population. Also, this can be due to more aggressive forms of cancer affecting this subgroup in these areas.^42^ Besides this, we could not consider neither the stage and grade at diagnosis to explain this difference more in detail.

Mortality trends are intrinsically limited by death certification validity. For this reason, we only included countries with WHO death certification data with over 90% death certification coverage, with good data quality, as declared by the WHO,^13^ and with a resident population of over 5 million, and over 20 for prediction analysis, in order to minimize issues of excessive variability. Predicted statistics for cancer mortality should be interpreted with caution, the selected prediction method is based on age‐specific joinpoint models, as such it is apt for short to medium term projections based on recent trends. Very recent changes in trends, occurring in the final 2–3 years of available data, and major changes in cohort effect trends could be difficult to detect with this prediction model. However, our previous European cancer mortality predictions, that have been running for over 10 years, have proved to be reliable and valid.^38^ Further, a previous age‐period‐cohort analysis displays monotonal descending cohort effects for the age groups and cohorts involved in these predictions, and comparing our previous prediction analysis on GC mortality published in 2014 with up to date registered data we found that the estimated projected ASMRs were accurate.^38^, ^43^ Broken line models, such as the joinpoint model used in this study, may also suffer from issues of overfitting. However, the NCI's joinpoint software is conservative in its model selection algorithms, and the frequentist permutation model selection method we use is the most conservative.^19^ The previous age‐period‐cohort analysis found an asymptotic trend in GC mortality in subjects born since the 1940 s in some Nordic and Western countries, foretelling the lack of progress in French middle‐aged women.^38^ One issue is whether misclassification on death certificates has changed over time: If it has remained the same, it would not affect the results on trends. In any case, the surveillance of trends in younger adults is needed to monitor mortality changes by age and to plan potential preventive interventions towards this subgroup.^42^

The analysis of 35–64 years truncated age group was performed for data quality and certification issues. That this truncated age group has higher mortality than the overall is expected, due to the weighting of the world standard population that gives younger age groups greater weights.

A major strength of this analysis is the high number of countries included for which real data were available, making our results more reliable than those based on estimates. The data provided in this manuscript are based on large countries, those reducing statistical fluctuation. Moreover, we could present results for both GC anatomical sites, namely cardia and noncardia.

## 5 CONCLUSION

Our findings confirm the declining mortality of GC in all countries with valid data, unfortunately this excludes China and other Asian, South American, and African countries of major interest for GC with lower data quality, limiting the representativeness of these results on a global population scale.^22^ The pattern of reduction is not homogeneous by geographical area and seems to follow the prevalence of *Hp* infection. The reduction in mortality rates is lower in US and EU countries, where the prevalence of infection has already reached a stable point, while high‐risk areas such as Eastern Europe and Asia have undergone an important decline in GC mortality, and will likely see a further decrease in 2025. Indeed, aggressive programs of screening and eradication of *Hp* infection may lead to a dramatic decrease in GC mortality in a relatively short time, in high‐risk populations.^44^

The distribution by anatomical site showed that the proportion of cardia and noncardia are getting close in many areas. Again, this may reflect the distribution of GC risk factors, with prevention of *Hp* infection and its eradication leading to a promising downscaling of noncardia GC in the EU and US, where cardia remains linked to lifestyle factors and obesity. The development of predictive models, the identification of high‐risk subjects, and the early “test‐and‐treat” of *Hp* at the population level may represent important preventive tools.

## AUTHOR CONTRIBUTIONS

**Giulia Collatuzzo:** Conceptualization (lead); formal analysis (supporting); validation (equal); writing – original draft (lead). **Claudia Santucci:** Data curation (supporting); formal analysis (lead); methodology (equal); writing – review and editing (lead). **Matteo Malvezzi:** Data curation (lead); formal analysis (equal); methodology (equal); writing – review and editing (lead). **Carlo La Vecchia:** Methodology (equal); project administration (supporting); resources (lead); supervision (supporting); visualization (equal); writing – review and editing (equal). **Paolo Boffetta:** Conceptualization (supporting); data curation (supporting); formal analysis (supporting); methodology (supporting); supervision (lead); validation (lead); writing – original draft (supporting); writing – review and editing (equal). **Eva Negri:** Funding acquisition (lead); project administration (lead); validation (equal); writing – review and editing (equal).

## CONFLICT OF INTEREST STATEMENT

All authors have declared no conflicts of interest.

## Supporting information


Data S1.

Table S1.
Table S2.
Table S3.
Table S4.
Table S5.

## Data Availability

The data that support the findings of this study are openly available in WHO database at http://www.who.int/healthinfo/statistics/mortality_rawdata/en/index.html.

## References

[1] Balakrishnan M , George R , Sharma A , Graham DY . Changing trends in stomach cancer throughout the world. Curr Gastroenterol Rep. 2017;19(8):36. 28730504 10.1007/s11894-017-0575-8 PMC6918953

[2] Global cancer observatory: cancer today. International Agency for Research on Cancer. Available at: https://gco.iarc.fr/today (Last accessed May 2022).

[3] Smyth EC , Nilsson M , Grabsch HI , van Grieken NC , Lordick F . Gastric cancer. Lancet. 2020;396(10251):635‐648. 32861308 10.1016/S0140-6736(20)31288-5

[4] Plummer M , Franceschi S , Vignat J , Forman D , de Martel C . Global burden of gastric cancer attributable to helicobacter pylori. Int J Cancer. 2015;136(2):487‐490. 24889903 10.1002/ijc.28999

[5] Sjomina O , Pavlova J , Niv Y , Leja M . Epidemiology of helicobacter pylori infection. Helicobacter. 2018;23(Suppl 1):e12514. 30203587 10.1111/hel.12514

[6] Lyons K , Le LC , Pham YT , et al. Gastric cancer: epidemiology, biology, and prevention: a mini review. Eur J Cancer Prev. 2019;28(5):397‐412. 31386635 10.1097/CEJ.0000000000000480

[7] Ward E , Jemal A , Cokkinides V , et al. Cancer disparities by race/ethnicity and socioeconomic status. CA Cancer J Clin. 2004;54(2):78‐93. 15061598 10.3322/canjclin.54.2.78

[8] https://www.wcrf.org/cancer‐trends/stomach‐cancer‐statistics/

[9] Franck C , Zimmermann N , Goni E , et al. Different prevalence of alarm, dyspeptic and reflux symptoms in patients with cardia and non‐cardia gastric cancer. J Gastrointestin Liver Dis. 2021;30(4):431‐437. 34752588 10.15403/jgld-3795

[10] Wilhelm TJ , Mothes H , Chiwewe D , Mwatibu B , Kähler G . Gastrointestinal endoscopy in a low budget context: delegating EGD to non‐physician clinicians in Malawi can be feasible and safe. Endoscopy. 2012;44(2):174‐176. 22068703 10.1055/s-0031-1291446

[11] World Health Organization Statistical Information System . WHO mortality database. World Health Organization; 2020. Available at: https://www.who.int/data/data‐collection‐tools/who‐mortality‐database (Last accessed March 2022).

[12] World Health Organization . International Statistical Classification of Disease and Related Health Problems: 10th Revision. World Health Organization; 1992.

[13] World Health Organization . WHO methods and data sources for country‐level causes of death 2000‐2019. Global Health Estimates Technical Paper WHO/DDI/DNA/GHE/2020.2. World Health Organization; 2020:7‐15.

[14] Pan American Health Organization (PAHO) .Health Information Platform for the Americas. Available at: https://www.paho.org/data/index.php/en/106‐cat‐data‐en/308‐poblacion‐nac‐en.html?showall=101.

[15] European Commission . EUROSTAT population database. Available at: https://ec.europa.eu/eurostat/web/main/data/database (Last accessed January 2022).

[16] United Nations DoEaSA, Population Division 2017 . World Population Prospects: the 2017 Revision, DVD Edition. United Nations; 2017.

[17] Doll R , Smith PG . Comparison between registries: age‐standardized rates. Vol. IV. IARC Sci Publ No. 42. In: JAH W , Muir CS , Shanmugaratnam K , Powell J , Peacham D , Whelan S , eds. Cancer Incidence in Five Continents. IARC; 1982:671‐675.

[18] National Cancer Institute . Joinpoint Regression Program, version 4.9.1. Available at: https://surveillance.cancer.gov/joinpoint/.

[19] Kim HJ , Fay MP , Feuer EJ , Midthune DN . Permutation tests for joinpoint regression with applications to cancer rates. Stat Med. 2000;19(3):335‐351. 10649300 10.1002/(sici)1097-0258(20000215)19:3<335::aid-sim336>3.0.co;2-z

[20] Clegg LX , Hankey BF , Tiwari R , Feuer EJ , Edwards BK . Estimating average annual per cent change in trend analysis. Stat Med. 2009;28(29):3670‐3682. 19856324 10.1002/sim.3733 PMC2843083

[21] Bray FCM , Mery L , Piñeros M , et al. Cancer Incidence in Five Continents, Vol. XI (Electronic Version). International Agency for Research on Cancer; 2017 Available from: https://ci5.iarc.fr

[22] Morgan E , Arnold M , Camargo MC , et al. The current and future incidence and mortality of gastric cancer in 185 countries, 2020‐40: a population‐based modelling study. EClinicalMedicine. 2022;47:101404. 35497064 10.1016/j.eclinm.2022.101404 PMC9046108

[23] Arnold M , Morgan E , Bardot A , et al. International variation in oesophageal and gastric cancer survival 2012‐2014: differences by histological subtype and stage at diagnosis (an ICBP SURVMARK‐2 population‐based study). Gut. 2022;71(8):1532‐1543. 34824149 10.1136/gutjnl-2021-325266

[24] Lordick F , Carneiro F , Cascinu S , et al. ESMO guidelines committee. Electronic address: clinicalguidelines@ESMO.Org. Gastric cancer: ESMO clinical practice guideline for diagnosis, treatment and follow‐up. Ann Oncol. 2022;33:1005‐1020. 35914639 10.1016/j.annonc.2022.07.004

[25] Asplund J , Kauppila JH , Mattsson F , Lagergren J . Survival trends in gastric adenocarcinoma: a population‐based study in Sweden. Ann Surg Oncol. 2018;25(9):2693‐2702. 29987609 10.1245/s10434-018-6627-y PMC6097732

[26] Colquhoun A , Arnold M , Ferlay J , Goodman KJ , Forman D , Soerjomataram I . Global patterns of cardia and non‐cardia gastric cancer incidence in 2012. Gut. 2015;64(12):1881‐1888. 25748648 10.1136/gutjnl-2014-308915

[27] Camargo MC , Freedman ND , Hollenbeck AR , Abnet CC , Rabkin CS . Height, weight, and body mass index associations with gastric cancer subsites. Gastric Cancer. 2014;17(3):463‐468. 24174008 10.1007/s10120-013-0312-4 PMC4007380

[28] Avgerinos KI , Spyrou N , Mantzoros CS , Dalamaga M . Obesity and cancer risk: emerging biological mechanisms and perspectives. Metabolism. 2019;92:121‐135. 30445141 10.1016/j.metabol.2018.11.001

[29] Lindblad M , Ye W , Lindgren A , Lagergren J . Disparities in the classification of esophageal and cardia adenocarcinomas and their influence on reported incidence rates. Ann Surg. 2006;243(4):479‐485. 16552198 10.1097/01.sla.0000205825.34452.43 PMC1448962

[30] Ekström AM , Signorello LB , Hansson LE , Bergström R , Lindgren A , Nyrén O . Evaluating gastric cancer misclassification: a potential explanation for the rise in cardia cancer incidence. J Natl Cancer Inst. 1999;91(9):786‐790. 10328109 10.1093/jnci/91.9.786

[31] Levi F , Te V‐C , Randimbison L , La Vecchia C . Re: evaluating gastric cancer misclassification: a potential explanation for the rise in cardia cancer incidence. JNCI: Journal of the National Cancer Institute. 1999;91(18):1585a‐1586a. 10.1093/jnci/91.18.1585a 10491439

[32] Chandrasoma P , Wickramasinghe K , Ma Y , DeMeester T . Adenocarcinomas of the distal esophagus and "gastric cardia" are predominantly esophageal carcinomas. Am J Surg Pathol. 2007;31(4):569‐575. doi:10.1097/01.pas.0000213394.34451.d2 17414104

[33] Taghavi N , Nasrollahzadeh D , Merat S , et al. Epidemiology of upper gastrointestinal cancers in Iran: a sub site analysis of 761 cases. World J Gastroenterol. 2007;13(40):5367‐5370. 17879408 10.3748/wjg.v13.i40.5367 PMC4171328

[34] Arnold M , Park JY , Camargo MC , Lunet N , Forman D , Soerjomataram I . Is gastric cancer becoming a rare disease? A global assessment of predicted incidence trends to 2035. Gut. 2020;69(5):823‐829. doi:10.1136/gutjnl-2019-320234 32001553 PMC8520492

[35] Carioli G , Bertuccio P , Levi F , et al. Cohort analysis of epithelial cancer mortality male‐to‐female sex ratios in the European Union, USA, and Japan. Int J Environ Res Public Health. 2020;17(15):5311. 32718003 10.3390/ijerph17155311 PMC7432705

[36] Heer EV , Harper AS , Sung H , Jemal A , Fidler‐Benaoudia MM . Emerging cancer incidence trends in Canada: the growing burden of young adult cancers. Cancer. 2020;126(20):4553‐4562. doi:10.1002/cncr.33050 32770762

[37] Lage J , Uedo N , Dinis‐Ribeiro M , Yao K . Surveillance of patients with gastric precancerous conditions. Best Pract Res Clin Gastroenterol. 2016;30(6):913‐922. 27938786 10.1016/j.bpg.2016.09.004

[38] Malvezzi M , Bonifazi M , Bertuccio P , et al. An age‐period‐cohort analysis of gastric cancer mortality from 1950 to 2007 in Europe. Ann Epidemiol. 2010;20(12):898‐905. 21074104 10.1016/j.annepidem.2010.08.013

[39] Roosendaal R , Kuipers EJ , Buitenwerf J , et al. Helicobacter pylori and the birth cohort effect: evidence of a continuous decrease of infection rates in childhood. Am J Gastroenterol. 1997;92(9):1480‐1482. 9317067

[40] Vaneckova P , Wade S , Weber M , et al. Birth‐cohort estimates of smoking initiation and prevalence in 20th century Australia: synthesis of data from 33 surveys and 385,810 participants. PLoS One. 2021;16(5):e0250824. 34019558 10.1371/journal.pone.0250824 PMC8139520

[41] Wang Z , Graham DY , Khan A , et al. Incidence of gastric cancer in the USA during 1999 to 2013: a 50‐state analysis. Int J Epidemiol. 2018;47(3):966‐975. 29672681 10.1093/ije/dyy055 PMC6005108

[42] Wang Z , El‐Serag HB , Thrift AP . Increasing incidence of advanced non‐cardia gastric cancers among younger Hispanics in the USA. Dig Dis Sci. 2021;66(5):1669‐1672. 32548813 10.1007/s10620-020-06397-x

[43] Ferro A , Peleteiro B , Malvezzi M , et al. Worldwide trends in gastric cancer mortality (1980‐2011), with predictions to 2015, and incidence by subtype. Eur J Cancer. 2014;50(7):1330‐1344. 24650579 10.1016/j.ejca.2014.01.029

[44] Asaka M , Kato M , Sakamoto N . Roadmap to eliminate gastric cancer with helicobacter pylori eradication and consecutive surveillance in Japan. J Gastroenterol. 2014;49(1):1‐8. 24162382 10.1007/s00535-013-0897-8 PMC3895201

