# Supplementary material for: Trends in gastric cancer mortality 1990–2019 in 36 countries worldwide, with predictions to 2025, and incidence, overall and by subtype
Source: Cancer Med. 2023 Feb 23;12(8):9912–25. doi: 10.1002/cam4.5685 (PMC10166912; doi:10.1002/cam4.5685)
Supplement: Supplementary file 1 — Data S1. [file CAM4-12-9912-s001.docx]

**SUPPLEMENTARY MATERIAL**

Table of Contents

[**Supplementary Table 1.** Age-standardized (world population) mortality rates and annual average deaths from gastric cancers per 100,000 person-years aged 35-64 in 2010-14 and 2015-19 for both sexes, along with the corresponding change in rates. 2](#_Toc121930576)

[**Supplementary Figure 1.** Bar plots of age-standardized mortality rates (ASMR) from gastric cancer among men and women adults aged 35-64 years per 100,000, in selected countries worldwide, in 2010-14 and 2015-19. 3](#_Toc121930577)

[**Supplementary Table 2**. Age-standardized (world population) mortality rates and annual average deaths from gastric cancers per 100,000 person-years aged 65 or more in 2010-14 and 2015-19 for both sexes, along with the corresponding change in rates. 4](#_Toc121930578)

[**Supplementary Figure 2.** Bar plots of age-standardized mortality rates (ASMR) from gastric cancer among older men and women aged 65 years or more per 100,000, in selected countries worldwide, in 2010-14 and 2015-19. 5](#_Toc121930579)

[**Supplementary Table 3.** Joinpoint analysis for gastric cancer, from 1990 to the last year available worldwide, for all ages and truncated at 35-64 years, men. 6](#_Toc121930580)

[**Supplementary Table 4.** Joinpoint analysis for gastric cancer, from 1990 to the last year available worldwide, for all ages and truncated at 35-64 years, women. 8](#_Toc121930581)

[**Supplementary Table 5**. Proportion and number of cardia and non-cardia gastric cancer cases, in selected countries among men. 10](#_Toc121930582)

[**Supplementary Table 6.** Proportion and number of cardia and non-cardia gastric cancer cases, in selected countries in women. 11](#_Toc121930583)

### **Supplementary Table 1.** Age-standardized (world population) mortality rates and annual average deaths from gastric cancers per 100,000 person-years aged 35-64 in 2010-14 and 2015-19 for both sexes, along with the corresponding change in rates.

|  | **Men** | | | | | **a** | **Women** | | | | |
| --- | --- | --- | --- | --- | --- | --- | --- | --- | --- | --- | --- |
|  | **Annual average deaths**  **2010-14** | **ASMR**  **2010-14** | **Annual average deaths**  **2015-19** | **ASMR**  **2015-19** | **% change 2015-19 vs 2010-14** |  | **Annual average deaths**  **2010-14** | **ASMR**  **2010-14** | **Annual average deaths**  **2015-19** | **ASMR**  **2015-19** | **% change 2015-19 vs 2010-14** |
| *Europe* |  |  |  |  |  |  |  |  |  |  |  |
| Austria | 119 | 6.38 | 118 | 5.76 | -9.7 |  | 73 | 3.85 | 71 | 3.49 | -9.4 |
| Belarus | 585 | 30.00 | 496 | 23.57 | -21.4 |  | 260 | 11.04 | 218 | 8.89 | -19.5 |
| Belgium | 120 | 4.88 | 114 | 4.56 | -6.6 |  | 50 | 2.08 | 51 | 2.07 | -0.5 |
| Czech Republic | 207 | 8.31 | 156 | 6.43 | -22.6 |  | 119 | 4.80 | 90 | 3.80 | -20.8 |
| Denmark | 73 | 5.73 | 75 | 5.92 | 3.3 |  | 38 | 3.05 | 32 | 2.58 | -15.4 |
| Finland | 71 | 5.45 | 61 | 4.79 | -12.1 |  | 44 | 3.42 | 37 | 2.92 | -14.6 |
| France | 815 | 5.92 | 787 | 5.58 | -5.7 |  | 318 | 2.27 | 310 | 2.13 | -6.2 |
| Germany | 1403 | 7.31 | 1328 | 6.50 | -11.1 |  | 763 | 4.00 | 704 | 3.54 | -11.5 |
| Greece | 179 | 7.51 | 188 | 7.95 | 5.9 |  | 100 | 4.05 | 95 | 3.78 | -6.7 |
| Hungary | 317 | 14.36 | 266 | 11.94 | -16.9 |  | 164 | 6.68 | 132 | 5.45 | -18.4 |
| Israel | 82 | 6.51 | 76 | 5.69 | -12.6 |  | 60 | 4.43 | 52 | 3.63 | -18.1 |
| Italy | 1112 | 8.24 | 997 | 7.12 | -13.6 |  | 643 | 4.59 | 602 | 4.11 | -10.5 |
| Netherlands | 202 | 5.14 | 173 | 4.35 | -15.4 |  | 122 | 3.20 | 95 | 2.39 | -25.3 |
| Norway | 44 | 4.00 | 51 | 4.40 | 10.0 |  | 26 | 2.49 | 23 | 2.19 | -12.0 |
| Poland | 1265 | 14.43 | 1063 | 12.06 | -16.4 |  | 515 | 5.60 | 470 | 5.13 | -8.4 |
| Portugal | 406 | 18.02 | 384 | 16.55 | -8.2 |  | 194 | 7.93 | 191 | 7.50 | -5.4 |
| Romania | 811 | 18.19 | 720 | 16.15 | -11.2 |  | 293 | 6.14 | 253 | 5.47 | -10.9 |
| Russian Federation | 8469 | 29.12 | 7178 | 23.19 | -20.4 |  | 4135 | 11.43 | 3465 | 9.17 | -19.8 |
| Spain | 842 | 8.62 | 780 | 7.46 | -13.5 |  | 416 | 4.15 | 414 | 3.86 | -7.0 |
| Sweden | 78 | 3.73 | 72 | 3.44 | -7.8 |  | 49 | 2.43 | 45 | 2.25 | -7.4 |
| Switzerland | 88 | 4.90 | 96 | 4.97 | 1.4 |  | 51 | 2.91 | 47 | 2.50 | -14.1 |
| Ukraine | 2480 | 27.66 | 1826 | 20.58 | -25.6 |  | 1206 | 10.79 | 881 | 8.15 | -24.5 |
| United Kingdom | 543 | 4.10 | 543 | 4.02 | -2.0 |  | 286 | 2.12 | 284 | 2.08 | -1.9 |
| EU-27 | 8961 | 9.08 | 8239 | 8.11 | -10.7 |  | 4356 | 4.31 | 4061 | 3.92 | -9.0 |
| *America* |  |  |  |  |  |  |  |  |  |  |  |
| Argentina | 647 | 9.96 | 657 | 9.44 | -5.2 |  | 331 | 4.71 | 324 | 4.30 | -8.7 |
| Brazil | 3560 | 11.63 | 3665 | 10.36 | -10.9 |  | 1813 | 5.26 | 1971 | 5.00 | -4.9 |
| Chile | 644 | 20.81 | 644 | 18.41 | -11.5 |  | 271 | 8.17 | 264 | 7.13 | -12.7 |
| Cuba | 167 | 7.03 | 175 | 6.55 | -6.8 |  | 92 | 3.80 | 98 | 3.70 | -2.6 |
| Mexico | 1126 | 7.14 | 1299 | 7.17 | 0.4 |  | 1040 | 6.12 | 1168 | 5.94 | -2.9 |
| Canada | 338 | 4.20 | 329 | 3.80 | -9.5 |  | 192 | 2.42 | 179 | 2.07 | -14.5 |
| USA | 2322 | 3.46 | 2332 | 3.34 | -3.5 |  | 1338 | 1.97 | 1384 | 1.99 | 1.0 |
| *Australasia* |  |  |  |  |  |  |  |  |  |  |  |
| Australia | 200 | 4.28 | 185 | 3.78 | -11.7 |  | 100 | 2.13 | 106 | 2.10 | -1.4 |
| Hong Kong SAR | 109 | 6.25 | 108 | 5.84 | -6.6 |  | 81 | 4.39 | 88 | 4.35 | -0.9 |
| Japan | 5609 | 17.65 | 3656 | 12.92 | -26.8 |  | 2549 | 8.47 | 1735 | 6.34 | -25.1 |
| Republic of Korea | 2179 | 19.73 | 1746 | 13.33 | -32.4 |  | 935 | 8.33 | 781 | 6.30 | -24.4 |

ASMR, Age standardized mortality rate.

### **Supplementary Figure 1.** Bar plots of age-standardized mortality rates (ASMR) from gastric cancer among men and women adults aged 35-64 years per 100,000, in selected countries worldwide, in 2010-14 and 2015-19.

### Supplementary Table 2. Age-standardized (world population) mortality rates and annual average deaths from gastric cancers per 100,000 person-years aged 65 or more in 2010-14 and 2015-19 for both sexes, along with the corresponding change in rates.

|  | **Men** | | | | | **a** | **Women** | | | | |
| --- | --- | --- | --- | --- | --- | --- | --- | --- | --- | --- | --- |
|  | **Annual average deaths**  **2010-14** | **ASMR**  **2010-14** | **Annual average deaths**  **2015-19** | **ASMR**  **2015-19** | **% change 2015-19 vs 2010-14** |  | **Annual average deaths**  **2010-14** | **ASMR**  **2010-14** | **Annual average deaths**  **2015-19** | **ASMR**  **2015-19** | **% change 2015-19 vs 2010-14** |
| *Europe* |  |  |  |  |  |  |  |  |  |  |  |
| Austria | 378 | 51.12 | 328 | 39.83 | -22.1 |  | 309 | 24.62 | 260 | 20.06 | -18.5 |
| Belarus | 623 | 143.23 | 555 | 117.33 | -18.1 |  | 551 | 54.62 | 449 | 40.87 | -25.2 |
| Belgium | 346 | 35.96 | 342 | 33.34 | -7.3 |  | 244 | 15.17 | 216 | 12.69 | -16.3 |
| Czech Republic | 453 | 61.11 | 435 | 49.62 | -18.8 |  | 347 | 27.93 | 316 | 23.23 | -16.8 |
| Denmark | 180 | 38.48 | 196 | 36.31 | -5.6 |  | 105 | 16.43 | 107 | 15.10 | -8.1 |
| Finland | 193 | 41.42 | 183 | 33.64 | -18.8 |  | 152 | 20.91 | 140 | 17.93 | -14.3 |
| France | 2128 | 38.37 | 2043 | 33.01 | -14.0 |  | 1325 | 13.89 | 1205 | 11.68 | -15.9 |
| Germany | 4251 | 51.30 | 3921 | 43.70 | -14.8 |  | 3436 | 26.26 | 2973 | 21.76 | -17.1 |
| Greece | 618 | 52.63 | 628 | 50.70 | -3.7 |  | 405 | 24.46 | 396 | 22.47 | -8.1 |
| Hungary | 626 | 91.96 | 576 | 77.96 | -15.2 |  | 540 | 41.40 | 483 | 35.36 | -14.6 |
| Israel | 4599 | 71.87 | 4476 | 63.95 | -11.0 |  | 3454 | 33.68 | 3268 | 29.72 | -11.8 |
| Italy | 630 | 46.38 | 593 | 36.46 | -21.4 |  | 412 | 21.10 | 355 | 16.43 | -22.1 |
| Netherlands | 145 | 36.52 | 147 | 33.38 | -8.6 |  | 114 | 19.49 | 90 | 13.27 | -31.9 |
| Norway | 2156 | 95.40 | 2193 | 83.11 | -12.9 |  | 1317 | 32.32 | 1330 | 29.24 | -9.5 |
| Poland | 995 | 103.73 | 981 | 93.14 | -10.2 |  | 731 | 46.94 | 705 | 41.50 | -11.6 |
| Portugal | 1460 | 103.52 | 1447 | 96.14 | -7.1 |  | 877 | 38.93 | 851 | 35.01 | -10.1 |
| Romania | 9863 | 153.56 | 9602 | 139.32 | -9.3 |  | 9596 | 63.71 | 8565 | 53.66 | -15.8 |
| Russian Federation | 2597 | 62.66 | 2490 | 54.36 | -13.2 |  | 1777 | 26.96 | 1649 | 23.31 | -13.5 |
| Spain | 291 | 31.06 | 264 | 25.69 | -17.3 |  | 214 | 17.39 | 176 | 13.49 | -22.4 |
| Sweden | 242 | 35.94 | 242 | 31.90 | -11.2 |  | 149 | 13.98 | 156 | 13.53 | -3.2 |
| Switzerland | 2833 | 120.07 | 2303 | 97.63 | -18.7 |  | 2177 | 44.68 | 1709 | 35.13 | -21.4 |
| Ukraine | 2447 | 42.63 | 2286 | 36.45 | -14.5 |  | 1466 | 17.76 | 1291 | 14.64 | -17.6 |
| United Kingdom | 23913 | 62.25 | 23159 | 55.17 | -11.4 |  | 17169 | 27.83 | 16040 | 24.40 | -12.3 |
| EU-27 | 378 | 51.12 | 328 | 39.83 | -22.1 |  | 309 | 24.62 | 260 | 20.06 | -18.5 |
| *America* |  |  |  |  |  |  |  |  |  |  |  |
| Argentina | 1156 | 60.15 | 1214 | 58.01 | -3.6 |  | 700 | 21.97 | 723 | 21.48 | -2.2 |
| Brazil | 5133 | 80.22 | 5558 | 69.71 | -13.1 |  | 2938 | 31.69 | 3145 | 27.56 | -13.0 |
| Chile | 1522 | 189.61 | 1530 | 158.24 | -16.5 |  | 821 | 65.90 | 810 | 53.80 | -18.4 |
| Cuba | 349 | 46.30 | 369 | 44.31 | -4.3 |  | 244 | 26.54 | 249 | 24.10 | -9.2 |
| Mexico | 1690 | 46.09 | 1863 | 43.73 | -5.1 |  | 1452 | 31.50 | 1573 | 30.57 | -3.0 |
| Canada | 809 | 30.18 | 891 | 27.42 | -9.1 |  | 553 | 14.54 | 553 | 13.07 | -10.1 |
| USA | 4285 | 20.16 | 4350 | 17.91 | -11.2 |  | 3142 | 10.27 | 3080 | 9.19 | -10.5 |
| *Australasia* |  |  |  |  |  |  |  |  |  |  |  |
| Australia | 525 | 30.95 | 522 | 25.82 | -16.6 |  | 316 | 13.90 | 312 | 11.99 | -13.7 |
| Hong Kong SAR | 284 | 52.18 | 310 | 50.11 | -4.0 |  | 182 | 24.71 | 175 | 20.05 | -18.9 |
| Japan | 26598 | 172.80 | 26104 | 145.98 | -15.5 |  | 14225 | 55.59 | 13802 | 47.79 | -14.0 |
| Republic of Korea | 3908 | 162.91 | 3430 | 108.08 | -33.7 |  | 2295 | 54.20 | 2010 | 35.80 | -33.9 |

ASMR, Age standardized mortality rate.

### **Supplementary Figure 2.** Bar plots of age-standardized mortality rates (ASMR) from gastric cancer among older men and women aged 65 years or more per 100,000, in selected countries worldwide, in 2010-14 and 2015-19.

### Supplementary Table 3. Joinpoint analysis for gastric cancer, from 1990 to the last year available worldwide, for all ages and truncated at 35-64 years, men.

| **Country** | **Years** | **APC1** | **Years** | **APC2** | **Years** | **APC3** | **Years** | **APC4** | **Years** | **APC5** | **Years** | **APC6** | **AAPC** |
| --- | --- | --- | --- | --- | --- | --- | --- | --- | --- | --- | --- | --- | --- |
| ***Europe*** |  |  |  |  |  |  |  |  |  |  |  |  |  |
| Belgium |  |  |  |  |  |  |  |  |  |  |  |  |  |
| All ages | 1990-2009 | -4.5* | 2009-2016 | -1.4 |  |  |  |  |  |  |  |  | -3.6* |
| Truncated 35-64 years | 1990-2016 | -3.1* |  |  |  |  |  |  |  |  |  |  | -3.1* |
| Czech Republic |  |  |  |  |  |  |  |  |  |  |  |  |  |
| All ages | 1990-2019 | -4.4* |  |  |  |  |  |  |  |  |  |  | -4.4* |
| Truncated 35-64 years | 1990-2019 | -4.1* |  |  |  |  |  |  |  |  |  |  | -4.1* |
| France |  |  |  |  |  |  |  |  |  |  |  |  |  |
| All ages | 1990-1994 | -4* | 1994-2016 | -2.6* |  |  |  |  |  |  |  |  | -2.8* |
| Truncated 35-64 years | 1990-1992 | -8.1* | 1992-2016 | -1.7* |  |  |  |  |  |  |  |  | -2.2* |
| Germany |  |  |  |  |  |  |  |  |  |  |  |  |  |
| All ages | 1990-1993 | -2.9* | 1993-2007 | -4.7* | 2007-2019 | -2.8* |  |  |  |  |  |  | -3.7* |
| Truncated 35-64 years | 1990-1995 | -3.4* | 1995-1998 | -6.8* | 1998-2019 | -2.7* |  |  |  |  |  |  | -3.2* |
| Greece |  |  |  |  |  |  |  |  |  |  |  |  |  |
| All ages | 1990-2018 | -1.8* |  |  |  |  |  |  |  |  |  |  | -1.8* |
| Truncated 35-64 years | 1990-2018 | -1.9* |  |  |  |  |  |  |  |  |  |  | -1.9* |
| Italy |  |  |  |  |  |  |  |  |  |  |  |  |  |
| All ages | 1990-2002 | -3.9* | 2002-2017 | -3.2* |  |  |  |  |  |  |  |  | -3.5* |
| Truncated 35-64 years | 1990-2003 | -4.4* | 2003-2017 | -3.2* |  |  |  |  |  |  |  |  | -3.7* |
| Netherlands |  |  |  |  |  |  |  |  |  |  |  |  |  |
| All ages | 1990-2018 | -4.3* |  |  |  |  |  |  |  |  |  |  | -4.3* |
| Truncated 35-64 years | 1990-2018 | -4.3* |  |  |  |  |  |  |  |  |  |  | -4.3* |
| Poland |  |  |  |  |  |  |  |  |  |  |  |  |  |
| All ages | 1990-1996 | -3.9* | 1996-2016 | -2.9* | 2016-2018 | -6.1* |  |  |  |  |  |  | -3.4* |
| Truncated 35-64 years | 1990-2005 | -3.9* | 2005-2015 | -2.5* | 2015-2018 | -6.7* |  |  |  |  |  |  | -3.7* |
| Portugal |  |  |  |  |  |  |  |  |  |  |  |  |  |
| All ages | 1990-2018 | -2.7* |  |  |  |  |  |  |  |  |  |  | -2.7* |
| Truncated 35-64 years | 1990-2018 | -2.5* |  |  |  |  |  |  |  |  |  |  | -2.5* |
| Romania |  |  |  |  |  |  |  |  |  |  |  |  |  |
| All ages | 1990-2002 | -0.7* | 2002-2018 | -2.4* |  |  |  |  |  |  |  |  | -1.6* |
| Truncated 35-64 years | 1990-2002 | -1.2* | 2002-2018 | -3* |  |  |  |  |  |  |  |  | -2.3* |
| Russian Federation |  |  |  |  |  |  |  |  |  |  |  |  |  |
| All ages | 1990-1993 | -1.4 | 1993-1996 | -5.1* | 1996-2003 | -2.7* | 2003-2018 | -3.6* |  |  |  |  | -3.3* |
| Truncated 35-64 years | 1990-1994 | -1.9* | 1994-1997 | -7* | 1997-2003 | -2.3* | 2003-2007 | -6.6* | 2007-2010 | -1.1 | 2010-2018 | -5.1* | -4.1* |
| Spain |  |  |  |  |  |  |  |  |  |  |  |  |  |
| All ages | 1990-2017 | -3.2* |  |  |  |  |  |  |  |  |  |  | -3.2* |
| Truncated 35-64 years | 1990-2017 | -3.4* |  |  |  |  |  |  |  |  |  |  | -3.4* |
| Sweden |  |  |  |  |  |  |  |  |  |  |  |  |  |
| All ages | 1990-2018 | -4* |  |  |  |  |  |  |  |  |  |  | -4* |
| Truncated 35-64 years | 1990-2018 | -3.4* |  |  |  |  |  |  |  |  |  |  | -3.4* |
| Ukraine |  |  |  |  |  |  |  |  |  |  |  |  |  |
| All ages | 1990-1992 | -0.7 | 1992-2007 | -3.1* | 2007-2012 | -2.1* | 2012-2019 | -4.6* |  |  |  |  | -3.2* |
| Truncated 35-64 years | 1990-1992 | 1.1 | 1992-2014 | -3.4* | 2014-2019 | -6.3* |  |  |  |  |  |  | -3.6* |
| United Kingdom |  |  |  |  |  |  |  |  |  |  |  |  |  |
| All ages | 1990-2010 | -4.9* | 2010-2016 | -3.1* |  |  |  |  |  |  |  |  | -4.5* |
| Truncated 35-64 years | 1990-2008 | -5.8* | 2008-2016 | -1.5* |  |  |  |  |  |  |  |  | -4.5* |
| EU-27 |  |  |  |  |  |  |  |  |  |  |  |  |  |
| All ages | 1990-1993 | -3.1* | 1993-2007 | -3.6* | 2007-2017 | -2.9* |  |  |  |  |  |  | -3.3* |
| Truncated 35-64 years | 1990-2003 | -3.7* | 2003-2017 | -2.8* |  |  |  |  |  |  |  |  | -3.2* |
| ***America*** |  |  |  |  |  |  |  |  |  |  |  |  |  |
| Argentina |  |  |  |  |  |  |  |  |  |  |  |  |  |
| All ages | 1990-2005 | -1.8* | 2005-2008 | -4.1 | 2008-2018 | -1.1* |  |  |  |  |  |  | -1.8* |
| Truncated 35-64 years | 1990-2018 | -1.9* |  |  |  |  |  |  |  |  |  |  | -1.9* |
| Brazil |  |  |  |  |  |  |  |  |  |  |  |  |  |
| All ages | 1990-2019 | -2.4* |  |  |  |  |  |  |  |  |  |  | -2.4* |
| Truncated 35-64 years | 1990-2019 | -2.3* |  |  |  |  |  |  |  |  |  |  | -2.3* |
| Chile |  |  |  |  |  |  |  |  |  |  |  |  |  |
| All ages | 1990-2000 | -1.1* | 2000-2018 | -2.9* |  |  |  |  |  |  |  |  | -2.3* |
| Truncated 35-64 years | 1990-2018 | -3* |  |  |  |  |  |  |  |  |  |  | -3* |
| Cuba |  |  |  |  |  |  |  |  |  |  |  |  |  |
| All ages | 1990-2017 | -1* |  |  |  |  |  |  |  |  |  |  | -1* |
| Truncated 35-64 years | 1990-2017 | -1* |  |  |  |  |  |  |  |  |  |  | -1* |
| Mexico |  |  |  |  |  |  |  |  |  |  |  |  |  |
| All ages | 1990-1994 | -0.2 | 1994-2008 | -2.3* | 2008-2013 | -3.8* | 2013-2017 | 0.3 |  |  |  |  | -1.9* |
| Truncated 35-64 years | 1990-1995 | 0.7 | 1995-2014 | -2.4* | 2014-2017 | 1.5 |  |  |  |  |  |  | -1.4* |
| Canada |  |  |  |  |  |  |  |  |  |  |  |  |  |
| All ages | 1990-2013 | -3.2* | 2013-2019 | -1.6* |  |  |  |  |  |  |  |  | -2.9* |
| Truncated 35-64 years | 1990-2019 | -2.8* |  |  |  |  |  |  |  |  |  |  | -2.8* |
| USA |  |  |  |  |  |  |  |  |  |  |  |  |  |
| All ages | 1990-2006 | -3.4* | 2006-2017 | -2.3* |  |  |  |  |  |  |  |  | -3* |
| Truncated 35-64 years | 1990-2006 | -3.6* | 2006-2017 | -1.3* |  |  |  |  |  |  |  |  | -2.6* |
| ***Australasia*** |  |  |  |  |  |  |  |  |  |  |  |  |  |
| Australia |  |  |  |  |  |  |  |  |  |  |  |  |  |
| All ages | 1990-2006 | -3.7* | 2006-2018 | -2.6* |  |  |  |  |  |  |  |  | -3.2* |
| Truncated 35-64 years | 1990-2006 | -3.6* | 2006-2018 | -1.3* |  |  |  |  |  |  |  |  | -2.6* |
| Hong Kong SAR |  |  |  |  |  |  |  |  |  |  |  |  |  |
| All ages | 1990-2017 | -3.1* |  |  |  |  |  |  |  |  |  |  | -3.1* |
| Truncated 35-64 years | 1990-2017 | -3.5* |  |  |  |  |  |  |  |  |  |  | -3.5* |
| Japan |  |  |  |  |  |  |  |  |  |  |  |  |  |
| All ages | 1990-1998 | -2* | 1998-2002 | -4.2* | 2002-2011 | -3* | 2011-2018 | -4.6* |  |  |  |  | -3.3* |
| Truncated 35-64 years | 1990-1996 | -3.2* | 1996-2013 | -4.2* | 2013-2018 | -7.5* |  |  |  |  |  |  | -4.6* |
| Republic of Korea |  |  |  |  |  |  |  |  |  |  |  |  |  |
| All ages | 1990-1994 | 1.7* | 1994-1999 | -5.9* | 1999-2002 | -1.5 | 2002-2010 | -6.5* | 2010-2019 | -7.7* |  |  | -5.2* |
| Truncated 35-64 years | 1990-2001 | -5.3* | 2001-2019 | -7.1* |  |  |  |  |  |  |  |  | -6.4* |

APC, annual percent change. AAPC, estimated average annual percent change.

*Significantly different from 0 (p<0.05).

### Supplementary Table 4. Joinpoint analysis for gastric cancer, from 1990 to the last year available worldwide, for all ages and truncated at 35-64 years, women.

| **Country** | **Years** | **APC1** | **Years** | **APC2** | **Years** | **APC3** | **Years** | **APC4** | **Years** | **APC5** | **Years** | **APC6** | **AAPC** |
| --- | --- | --- | --- | --- | --- | --- | --- | --- | --- | --- | --- | --- | --- |
| ***Europe*** |  |  |  |  |  |  |  |  |  |  |  |  |  |
| Belgium |  |  |  |  |  |  |  |  |  |  |  |  |  |
| All ages | 1990-2004 | -5.1* | 2004-2016 | -2.9* |  |  |  |  |  |  |  |  | -4.1* |
| Truncated 35-64 years | 1990-2016 | -2.9* |  |  |  |  |  |  |  |  |  |  | -2.9* |
| Czech Republic |  |  |  |  |  |  |  |  |  |  |  |  |  |
| All ages | 1990-2019 | -4* |  |  |  |  |  |  |  |  |  |  | -4* |
| Truncated 35-64 years | 1990-2019 | -3.1* |  |  |  |  |  |  |  |  |  |  | -3.1* |
| France |  |  |  |  |  |  |  |  |  |  |  |  |  |
| All ages | 1990-1996 | -4.5* | 1996-2016 | -2.6* |  |  |  |  |  |  |  |  | -3.1* |
| Truncated 35-64 years | 1990-1994 | -4.7* | 1994-2016 | -0.9* |  |  |  |  |  |  |  |  | -1.5* |
| Germany |  |  |  |  |  |  |  |  |  |  |  |  |  |
| All ages | 1990-2009 | -4.3* | 2009-2019 | -3.1* |  |  |  |  |  |  |  |  | -3.9* |
| Truncated 35-64 years | 1990-2019 | -3.2* |  |  |  |  |  |  |  |  |  |  | -3.2* |
| Greece |  |  |  |  |  |  |  |  |  |  |  |  |  |
| All ages | 1990-2018 | -2* |  |  |  |  |  |  |  |  |  |  | -2* |
| Truncated 35-64 years | 1990-2003 | -3.1* | 2003-2018 | 0 |  |  |  |  |  |  |  |  | -1.4* |
| Italy |  |  |  |  |  |  |  |  |  |  |  |  |  |
| All ages | 1990-2000 | -3.9* | 2000-2017 | -2.9* |  |  |  |  |  |  |  |  | -3.3* |
| Truncated 35-64 years | 1990-2017 | -2.6* |  |  |  |  |  |  |  |  |  |  | -2.6* |
| Netherlands |  |  |  |  |  |  |  |  |  |  |  |  |  |
| All ages | 1990-2014 | -2.9* | 2014-2018 | -8.2* |  |  |  |  |  |  |  |  | -3.7* |
| Truncated 35-64 years | 1990-2018 | -2.5* |  |  |  |  |  |  |  |  |  |  | -2.5* |
| Poland |  |  |  |  |  |  |  |  |  |  |  |  |  |
| All ages | 1990-2011 | -3.1* | 2011-2018 | -1.8* | 1990-2011 | -3.1* | 2011-2018 | -1.8* |  |  |  |  | -2.8* |
| Truncated 35-64 years | 1990-1999 | -3.7* | 1999-2018 | -1.9* | 1990-1999 | -3.7* | 1999-2018 | -1.9* |  |  |  |  | -2.5* |
| Portugal |  |  |  |  |  |  |  |  |  |  |  |  |  |
| All ages | 1990-2006 | -3.4* | 2006-2018 | -2.2* |  |  |  |  |  |  |  |  | -2.9* |
| Truncated 35-64 years | 1990-2018 | -2.4* |  |  |  |  |  |  |  |  |  |  | -2.4* |
| Romania |  |  |  |  |  |  |  |  |  |  |  |  |  |
| All ages | 1990-2018 | -2* |  |  |  |  |  |  |  |  |  |  | -2* |
| Truncated 35-64 years | 1990-2018 | -2.3* |  |  |  |  |  |  |  |  |  |  | -2.3* |
| Russian Federation |  |  |  |  |  |  |  |  |  |  |  |  |  |
| All ages | 1990-2009 | -3.3* | 2009-2018 | -4.2* |  |  |  |  |  |  |  |  | -3.6* |
| Truncated 35-64 years | 1990-1994 | -2.5* | 1994-1997 | -6* | 1997-2004 | -2.4* | 2004-2007 | -7* | 2007-2010 | -1.4 | 2010-2018 | -4.9* | -3.9* |
| Spain |  |  |  |  |  |  |  |  |  |  |  |  |  |
| All ages | 1990-2005 | -3.7* | 2005-2017 | -2.2* |  |  |  |  |  |  |  |  | -3* |
| Truncated 35-64 years | 1990-2005 | -3* | 2005-2017 | -1.5* |  |  |  |  |  |  |  |  | -2.3* |
| Sweden |  |  |  |  |  |  |  |  |  |  |  |  |  |
| All ages | 1990-2018 | -3.5* |  |  |  |  |  |  |  |  |  |  | -3.5* |
| Truncated 35-64 years | 1990-2018 | -3.2* |  |  |  |  |  |  |  |  |  |  | -3.2* |
| Ukraine |  |  |  |  |  |  |  |  |  |  |  |  |  |
| All ages | 1990-1992 | -0.6 | 1992-1995 | -5.1* | 1995-2012 | -2.8* | 2012-2019 | -4.7* |  |  |  |  | -3.3* |
| Truncated 35-64 years | 1990-2012 | -2.9* | 2012-2019 | -5.3* |  |  |  |  |  |  |  |  | -3.4* |
| United Kingdom |  |  |  |  |  |  |  |  |  |  |  |  |  |
| All ages | 1990-2007 | -4.5* | 2007-2016 | -3.1* |  |  |  |  |  |  |  |  | -4* |
| Truncated 35-64 years | 1990-2008 | -4.6* | 2008-2016 | 0.1 |  |  |  |  |  |  |  |  | -3.2* |
| EU-27 |  |  |  |  |  |  |  |  |  |  |  |  |  |
| All ages | 1990-2006 | -3.6* | 2006-2017 | -2.8* |  |  |  |  |  |  |  |  | -3.3* |
| Truncated 35-64 years | 1990-2005 | -3.2* | 2005-2017 | -2.1* |  |  |  |  |  |  |  |  | -2.7* |
| ***America*** |  |  |  |  |  |  |  |  |  |  |  |  |  |
| Argentina |  |  |  |  |  |  |  |  |  |  |  |  |  |
| All ages | 1990-2003 | -2.6* | 2003-2018 | -1* |  |  |  |  |  |  |  |  | -1.8* |
| Truncated 35-64 years | 1990-2018 | -0.9* |  |  |  |  |  |  |  |  |  |  | -0.9* |
| Brazil |  |  |  |  |  |  |  |  |  |  |  |  |  |
| All ages | 1990-2019 | -1.9* |  |  |  |  |  |  |  |  |  |  | -1.9* |
| Truncated 35-64 years | 1990-2000 | -2.3* | 2000-2019 | -1* |  |  |  |  |  |  |  |  | -1.4* |
| Chile |  |  |  |  |  |  |  |  |  |  |  |  |  |
| All ages | 1990-2018 | -2.7* |  |  |  |  |  |  |  |  |  |  | -2.7* |
| Truncated 35-64 years | 1990-2018 | -2.3* |  |  |  |  |  |  |  |  |  |  | -2.3* |
| Cuba |  |  |  |  |  |  |  |  |  |  |  |  |  |
| All ages | 1990-2017 | -0.7* |  |  |  |  |  |  |  |  |  |  | -0.7* |
| Truncated 35-64 years | 1990-2017 | -1.3* |  |  |  |  |  |  |  |  |  |  | -1.3* |
| Mexico |  |  |  |  |  |  |  |  |  |  |  |  |  |
| All ages | 1990-2005 | -1.6* | 2005-2013 | -2.9* | 2013-2017 | 0.1 |  |  |  |  |  |  | -1.7* |
| Truncated 35-64 years | 1990-1999 | 0 | 1999-2017 | -1.9* |  |  |  |  |  |  |  |  | -1.3* |
| Canada |  |  |  |  |  |  |  |  |  |  |  |  |  |
| All ages | 1990-2019 | -2.6* |  |  |  |  |  |  |  |  |  |  | -2.6* |
| Truncated 35-64 years | 1990-2019 | -2* |  |  |  |  |  |  |  |  |  |  | -2* |
| USA |  |  |  |  |  |  |  |  |  |  |  |  |  |
| All ages | 1990-2009 | -2.5* | 2009-2017 | -1.4* |  |  |  |  |  |  |  |  | -2.2* |
| Truncated 35-64 years | 1990-2008 | -2.1* | 2008-2017 | 0.2 |  |  |  |  |  |  |  |  | -1.3* |
| ***Australasia*** |  |  |  |  |  |  |  |  |  |  |  |  |  |
| Australia |  |  |  |  |  |  |  |  |  |  |  |  |  |
| All ages | 1990-2018 | -2.8* |  |  |  |  |  |  |  |  |  |  | -2.8* |
| Truncated 35-64 years | 1990-2018 | -2.2* |  |  |  |  |  |  |  |  |  |  | -2.2* |
| Hong Kong SAR |  |  |  |  |  |  |  |  |  |  |  |  |  |
| All ages | 1990-2017 | -2.8* |  |  |  |  |  |  |  |  |  |  | -2.8* |
| Truncated 35-64 years | 1990-2017 | -2.2* |  |  |  |  |  |  |  |  |  |  | -2.2* |
| Japan |  |  |  |  |  |  |  |  |  |  |  |  |  |
| All ages | 1990-1999 | -3.3* | 1999-2018 | -3.9* |  |  |  |  |  |  |  |  | -3.7* |
| Truncated 35-64 years | 1990-1999 | -3.5* | 1999-2014 | -4.4* | 2014-2018 | -7.3* |  |  |  |  |  |  | -4.5* |
| Republic of Korea |  |  |  |  |  |  |  |  |  |  |  |  |  |
| All ages | 1990-1993 | 2.8 | 1993-2005 | -5.4* | 2005-2019 | -6.8* |  |  |  |  |  |  | -5.2* |
| Truncated 35-64 years | 1990-2019 | -6.1* |  |  |  |  |  |  |  |  |  |  | -6.1* |

APC, annual percent change. AAPC, estimated average annual percent change.

*Significantly different from 0 (p<0.05).

### Supplementary Table 5. Proportion and number of cardia and non-cardia gastric cancer cases, in selected countries among men.

|  | **Cardia cases**  **(C16.0)** | **Non-cardia cases**  **(C16.1-6)** | **a** | **Proportion**  **of cardia cases (%)** |  |
| --- | --- | --- | --- | --- | --- |
| ***Europe*** |  |  |  |  |  |
| Austria | 742 | 2946 |  | 20.1 |  |
| Belarus | 628 | 5491 |  | 10.3 |  |
| Belgium | 1560 | 2751 |  | 36.2 |  |
| Czech Republic | 767 | 3132 |  | 19.7 |  |
| Denmark | 1004 | 616 |  | 62.0 |  |
| France | 1223 | 1976 |  | 38.2 |  |
| Germany | 9256 | 17,506 |  | 34.6 |  |
| Israel | 483 | 1553 |  | 23.7 |  |
| Italy | 1626 | 8081 |  | 16.8 |  |
| Netherlands | 2094 | 3485 |  | 37.5 |  |
| Norway | 431 | 859 |  | 33.4 |  |
| Russian Federation | 717 | 4217 |  | 14.5 |  |
| Spain | 586 | 2341 |  | 20.0 |  |
| Switzerland | 488 | 863 |  | 36.1 |  |
| Ukraine | 4307 | 20,810 |  | 17.1 |  |
| United Kingdom | 8133 | 14,536 |  | 35.9 |  |
| ***America*** |  |  |  |  |  |
| Argentine | 80 | 1196 |  | 6.3 |  |
| Chile | 101 | 708 |  | 12.5 |  |
| Canada | 2736 | 3994 |  | 40.7 |  |
| USA | 26,649 | 36,296 |  | 42.3 |  |
| ***Australasia*** |  |  |  |  |  |
| Australia | 2445 | 3759 |  | 39.4 |  |
| Japan | 6746 | 48,118 |  | 12.3 |  |
| Republic of Korea | 4729 | 65,437 |  | 6.7 |  |

Data refer to registries or groups of registries from the same country, with data available in Cancer Incidence in Five Continents (CI5)-XI, with available incidence data for 2008-2012, i.e., Austria, Belarus, Belgium, Czech Republic, Denmark, France (Calvados, Doubs, Gironde, Haut-Rhin, Hérault, Isère, Lille-Métropole, Loire-Atlantique, Somme, Tarn, Territoire de Belfort, Vendée), Germany (Bavaria, Bremen, Hamburg, Lower Saxony, Munich, North Rhine-Westphalia, Rhineland-Palatinate, Saarland, Schleswig-Holstein), Israel, Italy (Aosta Valley, Bergamo, Biella, Catania, Messina and Enna, Latina, Milan, Modena, Monza, Naples, Nuoro, Palermo, Parma, Ragusa and Caltanissetta, Reggio Emilia, Romagna, Sondrio, Syracuse, Turin, Varese), Netherlands, Norway, Russian Federation (Arkhangelsk, Chelyabinsk, Karelia, Samara), Spain (Basque Country, Castellón, Girona, Granada, La Rioja, Tarragona), Switzerland (Fribourg, Geneva, Graubünden and Glarus, Neuchâtel, St Gall-Appenzell, Ticino, Valais, Vaud, Switzerland, Zurich), Ukraine, United Kingdom, Chile (Bío Bío Province, Valdivia), Canada (Alberta, British Columbia, Manitoba, New Brunswick, Newfoundland and Labrador, Northwest Territories, Nova Scotia, Nunavut, Ontario, Prince Edward Island, Saskatchewan, Yukon), USA, Australia, Japan (Aichi Prefecture, Fukui Prefecture, Hiroshima Prefecture, Nagasaki Prefecture, Niigata Prefecture, Osaka Prefecture, Tochigi Prefecture, Yamagata Prefecture), and Republic of Korea.

### Supplementary Table 6. Proportion and number of cardia and non-cardia gastric cancer cases, in selected countries in women.

|  | **Cardia cases**  **(C16.0)** | **Non-cardia cases**  **(C16.1-6)** | **a** | **Proportion of**  **cardia cases (%)** |
| --- | --- | --- | --- | --- |
| ***Europe*** |  |  |  |  |
| Austria | 261 | 2518 |  | 9.4 |
| Belarus | 305 | 4133 |  | 6.9 |
| Belgium | 392 | 2185 |  | 15.2 |
| Czech Republic | 305 | 2432 |  | 11.1 |
| Denmark | 271 | 502 |  | 35.1 |
| France | 304 | 1441 |  | 17.4 |
| Germany | 2848 | 15,174 |  | 15.8 |
| Israel | 193 | 1174 |  | 14.1 |
| Italy | 498 | 6276 |  | 7.4 |
| Netherlands | 670 | 2437 |  | 21.6 |
| Norway | 146 | 694 |  | 17.4 |
| Russian Federation | 439 | 3505 |  | 11.1 |
| Spain | 138 | 1548 |  | 8.2 |
| Switzerland | 139 | 655 |  | 17.5 |
| Ukraine | 1963 | 14,379 |  | 12.0 |
| United Kingdom | 2538 | 9687 |  | 20.8 |
| ***America*** |  |  |  |  |
| Argentine | 23 | 627 |  | 3.5 |
| Chile | 28 | 328 |  | 7.9 |
| Canada | 830 | 2860 |  | 22.5 |
| USA | 7520 | 29,666 |  | 20.2 |
| ***Australasia*** |  |  |  |  |
| Australia | 755 | 2463 |  | 23.5 |
| Japan | 2147 | 23,479 |  | 8.4 |
| Republic of Korea | 1545 | 30,815 |  | 4.8 |

Data refer to registries or groups of registries from the same country, with data available in Cancer Incidence in Five Continents (CI5)-XI, with available incidence data for 2008-2012, i.e., Austria, Belarus, Belgium, Czech Republic, Denmark, France (Calvados, Doubs, Gironde, Haut-Rhin, Hérault, Isère, Lille-Métropole, Loire-Atlantique, Somme, Tarn, Territoire de Belfort, Vendée), Germany (Bavaria, Bremen, Hamburg, Lower Saxony, Munich, North Rhine-Westphalia, Rhineland-Palatinate, Saarland, Schleswig-Holstein), Israel, Italy (Aosta Valley, Bergamo, Biella, Catania, Messina and Enna, Latina, Milan, Modena, Monza, Naples, Nuoro, Palermo, Parma, Ragusa and Caltanissetta, Reggio Emilia, Romagna, Sondrio, Syracuse, Turin, Varese), Netherlands, Norway, Russian Federation (Arkhangelsk, Chelyabinsk, Karelia, Samara), Spain (Basque Country, Castellón, Girona, Granada, La Rioja, Tarragona), Switzerland (Fribourg, Geneva, Graubünden and Glarus, Neuchâtel, St Gall-Appenzell, Ticino, Valais, Vaud, Switzerland, Zurich), Ukraine, United Kingdom, Chile (Bío Bío Province, Valdivia), Canada (Alberta, British Columbia, Manitoba, New Brunswick, Newfoundland and Labrador, Northwest Territories, Nova Scotia, Nunavut, Ontario, Prince Edward Island, Saskatchewan, Yukon), USA, Australia, Japan (Aichi Prefecture, Fukui Prefecture, Hiroshima Prefecture, Nagasaki Prefecture, Niigata Prefecture, Osaka Prefecture, Tochigi Prefecture, Yamagata Prefecture), and Republic of Korea.
